# Editorial Items

**Published:** 1871-07

**Authors:** 


If gentlemen who have taken the position assumed by the Faculty of the Atlanta Medical College, and used the means *they* have employed to maintain such position, and to carry out their objects,—endorsed by a Board of Trustees of their own selection, (who are willing to establish the principle that those who are wrong are to be sustained and those who are right condemned) we presume that few first-class gentlemen will be deceived into a professorship.

We are glad to learn that the editors of the *Atlanta Medical and Surgical Journal* are "*wholly*" and "*solely*" responsible for all that has appeared in a so-called "statement of facts." We feel certain that none of "our friends" would be responsible for such "facts." The time may come when *they* will rather wish they had not so cheerfully "assumed" the responibility. They may have occasion to again "condemn ourselves."

We return our thanks to Dr. F. D. Thurman, proprietor of Solitude Strawberry Plantation, Atlanta, Ga., for a bottle of his superior strawberry wine. It is very fine, and has been, we are informed, pronounced by the best judges, to be equal, and in some respects superior to the purest champagne. As a wine for medical purposes it will be found excellent, and can be relied upon as being pure.

The Bellevue Hospital Medical College, New York, requires all young men before matriculating, to come endorsed as possessing good moral character. This we sanction and approve. If this is necessary to enter a medical college, why is it that, after having obtained a diploma, graduates may resort to tricks, and "aid and abet" every fellow who would pull down the profession to his own unprofessional level? Such men slide from a higher to a lower region, and are unworthy the name of Doctor.

